# Seroprevalance of antibodies specific for severe fever with thrombocytopenia syndrome virus and the discovery of asymptomatic infections in Henan Province, China

**DOI:** 10.1371/journal.pntd.0007242

**Published:** 2019-11-25

**Authors:** Yanhua Du, Ningning Cheng, Yi Li, Haifeng Wang, Aiguo You, Jia Su, Yifei Nie, Hongxia Ma, Bianli Xu, Xueyong Huang

**Affiliations:** 1 Henan Center for Disease Control and Prevention, Zhengzhou, China; 2 Henan Key Laboratory of Pathogenic Microorganisms, Zhengzhou, China; 3 Kaifeng Center for Disease Control and Prevention, Kaifeng, China; 4 Henan Collaborative Innovation Center of Molecular Diagnosis and Laboratory Medicine, Xinxiang, China; Faculty of Science, Ain Shams University (ASU), EGYPT

## Abstract

**Background:**

Severe fever with thrombocytopenia syndrome (SFTS) is a severe emerging disease caused by SFTS virus (SFTSV), and the geographical distribution of SFTS has been increasing throughout China in recent years. To assess SFTSV-specific antibody seroprevalence, a cross-sectional study was conducted for healthy people in high SFTS endemic areas of Henan province in 2016.

**Methods:**

This study used a stratified random sampling method to select 14 natural villages as the investigation sites. From April to May 2016, participants completed a questionnaire survey and serum samples were collected. All serum samples were subjected to ELISA to detect SFTSV-specific IgM and IgG. All IgM-positive samples were further tested by real-time RT-PCR, and isolation of virus from serum was attempted. Any participant who was IgM-positive was followed up with a month later to confirm health status.

**Results:**

In total, 1463 healthy people participated in this study. The average seropositive rates for SFTSV-specific IgG and IgM were 10.46% (153/1463) and 0.82% (12/1463), respectively. IgM was detected in 12 individuals, and SFTSV RNA was detected in six of them. Virus was isolated from five of the six SFTSV RNA-positive individuals, and phylogenetic analyses revealed that all five isolates belonged to SFTSV group A. No IgM-positive participants exhibited any symptoms or other signs of illness at the one-month follow up.

**Conclusions:**

This study identified a relatively high incidence of SFTSV-specific antibody seropositivity in healthy people in Xinyang city. Moreover, our data provide the first evidence for asymptomatic SFTSV infections, which may have significant implications for SFTS outbreak control.

## Author summary

Severe fever with thrombocytopenia syndrome (SFTS) is a severe emerging infectious disease caused by SFTS virus (SFTSV) that was first discovered in rural areas of China. Henan province has had the largest number of SFTS cases in China every year since the disease was discovered, however, seropositivity for SFTSV-specific antibodies in healthy people in this region is still not clear. To address this issue, a cross-sectional survey was performed in high endemic areas from April to May 2016. The results showed that SFTSV seroprevalence was relatively high and possibly increasing. Notably, SFTSV RNA, as well as virus itself, was isolated from specimens obtained from healthy people. This study confirmed there are asymptomatic SFTSV infections in humans, and it is the first to report SFTSV isolation from healthy people.

## Introduction

Severe fever with thrombocytopenia syndrome (SFTS) is a tick-borne emerging infectious disease that first appeared in eastern China in 2006 [1–3]. Since then, SFTS cases have been reported in almost 25 provinces of China as well as other countries, including Japan, Korea, and Vietnam [4–6]. The major clinical features of disease include fever, thrombocytopenia, leukocytopenia, gastrointestinal symptoms, and neurological symptoms, as well as other, less specific clinical manifestations [7,8]. The average case fatality rate of SFTS was about 30% when this disease was firstly reported [1]. In 2009, SFTS virus (SFTSV) was identified from a patient located in Xinyang, Henan, China as the etiologic agent of SFTS [9]. The public health threat posed by SFTSV was highlighted in 2016 and 2017, the World Health Organization listed the virus as priority pathogen requiring urgent attention [10].

SFTSV is prevalent mainly in seven central-eastern provinces of China including Henan, Hubei, Anhui, Jiangsu, Zhejiang, Shandong, and Liaoning. According to the National Notifiable Diseases Surveillance System (an administrative database developed by China CDC), more than 85% of SFTS cases were reported in rural regions of these seven provinces, with the highest number reported in Henan province since 2010 [11,12]. Xinyang city of Henan province located around Dabie mountain is a high endemic area, where more than 95% of SFTS cases come from Henan province [13]. Therefore, a cross-sectional study was performed in rural areas of Xinyang city to identify the actual seroprevalence of SFTSV.

## Methods

### Study design

A cross-sectional investigation was conducted in Xinyang city by random cluster sampling. The city was divided into 10 administrative counties/districts. First, one county (Xin) and one district (Pingqiao) were selected and then one town was selected from each (Balifan and Pengjiawan, respectively). From these two towns, fourteen natural villages that had previously reported cases of SFTS were selected and healthy individuals from these villages were recruited for the study.

The survey participants were selected using strict criteria. For the purposes of this study, a healthy person was defined as someone who had lived in the area for more than 1 year, was aged 2 years or older, and had no history of fever or other discomfort for the two weeks prior to enrollment. People who had been diagnosed with SFTS in the past were excluded from the study. Previous studies have shown SFTSV seroprevalence to range between 7.2% and 10.5% in healthy people with no reported symptoms associated with SFTS [14]. Thus, assuming a 7% incidence of SFTS, the minimum sample size required for 80% power and a two tailed 5% level of significance in this study was calculated to be 1276.

### Field Investigation and sample collection

The survey was carried out from April to May 2016. A questionnaire was used to collect information on name, gender, age, occupation, home address, and history of fever or SFTS. Serum samples were collected from survey subjects who met the inclusion criteria. All samples were transported frozen to the pathogen laboratory of the Henan Center for Disease Control and Prevention (Henan CDC).

### Indirect Enzyme-linked Immunosorbent Assays (ELISA)

For the indirect IgG ELISA, 96-well plates were coated with 50 ng recombinant SFTSV nucleoprotein per well overnight at 4°C. Recombinant SFTSV nucleoprotein was expressed and purified as previously described [15]. Plates were washed three times with wash buffer (0.01M PBS, 0.05% Tween 20). Serum samples were diluted at 1:400 in 5% nonfat milk with PBS-T, and 50 μL of each sample were added to each well. Plates were incubated at 37°C for 1 hour and then washed with wash buffer before wells were treated with 1:30,000 diluted horseradish peroxidase-conjugated goat anti-human IgG (American Qualex, California, USA) at 37°C for 1 hour. After washing, 100 μL H_2_O_2_-ABTS substrate (Kirkegaard & Perry Laboratories Inc., Gaithersburg, MD) was added to each well, and plates were incubated for 30 min at 37°C. Absorbance was measured at 405 nm (including negative and blank controls). The test results were determined to be valid if the criteria for the positive control and the negative controls were fulfilled. Calculation of the cut-off value was the mean absorbance value for negative controls times 2.1; if the mean absorbance value for negative controls was lower than 0.05, then the value 0.05 was used. Positive/negative determination of samples was performed using the cut-off value.

The procedure for the indirect IgM ELISAs was similar to that for the indirect IgG ELISAs, except that the detection of bound IgM was done with 1:10,000 diluted horseradish peroxidase-conjugated goat anti-human IgM (American Qualex, Califonia, USA).

### Detection of SFTSV by real-time RT-PCR

Total RNA was extracted from SFTSV-IgM positive samples using a QIAamp viral RNA mini Kit (Qiagen, Germany), following the manufacturer’s instructions. The real-time RT-PCR assay using PCR Diagnostic Kit for SFTSV RNA (BGI-GBI, China) was performed as previously described [16]. Data were analyzed using the software supplied by the manufacturer.

### Virus isolation and sequencing

SFTSV RT-PCR positive serum samples (100 μL) were used to inoculate Vero cells for virus isolation. Following inoculation, Vero cell monolayers were incubated for 7–10 days at 37°C and 5% CO_2_ in MEM/1% fetal calf serum and monitored daily for cytopathic effect (CPE). Virus-positive cells were identified over the course of three passages by the presence of CPE as well as real-time RT-PCR, as previously described [17]. Samples were judged as negative and excluded if they remained negative for CPE and real-time RT-PCR even after three passages. The whole genome sequence of SFTSV isolates was amplified using primers described previously [18,19]. PCR products were sent to Jinsirui Biotech Co., Ltd (Nanjing, China) for DNA sequencing using an automated ABI 3730 DNA Sequencer. In order to confirm results of real-time RT-PCR, the PCR products were also sent to Takara Biomedical Technology Co., Ltd. (Beijing, China) for DNA sequencing by using plasmid cloning technique.

### Phylogenetic analysis

The genomes of SFTSV isolates were compiled using the SeqMan program in the DNAStar package, version 7.1.0. (Lasergene, DNAStar, Inc. Madison, WI, USA). Molecular phylogenetic analysis was conducted using the maximum likelihood (ML) method based on the Kimura 2-parameter model in MEGA 5.05 software (http://www.megasoftware.net/) [20]. Thirty SFTSV sequences obtained from GenBank were analyzed together with our newly generated SFTSV genomes, and phylogenetic trees were constructed in order to understand the evolutionary relationships of SFTSV isolates.

### Statistical analysis

All epidemiologic and laboratory data were double-entered with the EpiData 3.1 software. All statistical analyses were performed using SAS v9.13 (SAS Institute Inc., Cary, NC). Count data was analyzed by χ2 test or Fisher’s exact test. A difference was considered significant when the P-value was less than 0.05.

### Ethics statement

This research was approved by the Institutional Review Board at the Center for Disease Control and Prevention of Henan Province (NO.2016-KY-002-02). The methods were carried out in accordance with the principles of the Declaration of Helsinki. All adult participants signed the written informed consent and agreed to use their serum samples for research. In the case of child participants, a parent or legal guardian signed the written informed consent on their child’s behalf.

## Results

### Survey subjects and serum samples

A total of 1463 healthy persons were surveyed in high SFTS epidemic areas: 768 from Xin county and 695 from Pingqiao district. Among the survey subjects, 409 were males and 1054 were females, with a male–female sex ratio of 0.39. The median age of the survey subjects was 60 years (range: 2–92). Serum samples were collected from every survey subject.

### SFTSV IgG and IgM prevalence and isolation of virus

All serum samples were tested for SFTSV IgG and IgM by indirect ELISA. From the 1463 individuals, 153 were positive for IgG and 12 were positive for IgM, giving seropositivity rates of 10.46% (153/1463) and 0.82% (12/1463), respectively. The median age of these individuals was 60 years (range, 32–73), and the sex ratio was 1 to 2 (4 males and 8 females). There were no statistically significant differences in IgG or IgM seropositivity rates between gender or among different age groups (Table 1). Among the 12 IgM-positive samples, six were positive for SFTSV RNA by real-time RT-PCR and we isolated SFTSV from five (Table 2). Notably, the viral RNA levels increased over three passages for these five isolates, indicating a productive infection (Table 2). The 12 IgM-positive individuals were followed up with one month after initial sampling. None reported a fever or any other typical clinical presentation associated with SFTS, including headache, muscle aches, nausea, or vomiting, either at the time of follow-up or during the preceding one-month period. Serum samples taken at the follow-up visit were negative for SFTSV RNA and IgM but positive for IgG. Together these data suggest that these 12 individuals experienced an asymptomatic SFTSV infection.

**Table 1:** Demographic characteristic of SFTSV IgG and IgM seropositive in healthy people.

Variable	No.	Positive for IgG (ratio, positive/total)	Positive for IgM (ratio, positive/total)	Constituent ratio
**Gender**				
Males	409	42(10.27%)	4(0.98%)	27.96%
Females	1054	111(10.53%)	8(0.76%)	72.04%
Total	1463			
χ2		0.022	0.009*	
P		0.883	0.925	
**Age (years old)**				
≤20	36	2(5.56%)	0(0.00%)	2.46%
21~30	19	1(5.26%)	0(0.00%)	1.30%
31~40	59	6(10.17%)	2(3.39%)	4.03%
41~50	264	16(6.06%)	3(1.14%)	18.05%
51~60	390	48(12.31%)	1(0.26%)	26.66%
61~70	437	54(12.36%)	4(0.92%)	29.87%
>70	258	26(10.08%)	2(0.78%)	17.63%
Total	1463	153(10.46%)	12(0.82%)	
χ2		10.076	7.147	
P		0.121	0.307	

**Table 2:** Results of ELISA and real-time RT-PCR in 12 IgM seropositive healthy persons.

Persons No.	Original No.	Sex	Age	Uncultured serum specimens				Cultured serum specimens			
				ELISA	ELISA	Real-time PCR		CPE	Real-time PCR		
				IgG	IgM	CT value	Sequencing		P1	P2	P3
1	ZG028	M	55	-	+	49.12	-	+	21.65	17.45	13.88
2	ZG085	M	69	-	+	-	/	-	-	-	-
3	PZ090	F	72	-	+	-	/	-	-	-	-
4	BL075	F	32	-	+	-	/	-	-	-	-
5	BL125	F	69	-	+	-	/	-	-	-	-
6	BL136	F	45	-	+	38.62	-	-	-	-	-
7	BL238	M	69	-	+	-	/	-	-	-	-
8	BL282	F	73	-	+	42.26	+	+	23.43	18.66	12.94
9	BL316	F	45	-	+	-	/	-	-	-	-
10	SW131	F	64	-	+	39.81	+	+	20.34	15.87	13.88
11	SW228	M	50	-	+	41.42	+	+	22.60	17.23	14.20
12	SW249	F	40	+	+	38.94	+	+	19.89	15.78	13.62

### Molecular characterization of SFTSV strains

The complete genomes of the five SFTSV isolates obtained from participant serum samples were successfully sequenced, and all sequences were submitted to GenBank (Accession numbers: MF045948- MF045952, MF045955- MF045959, MF045962- MF045965 and MF045968). The nucleotide sequences of the five SFTSV isolates were closely related to each other, with 99.95% to 99.98% nucleotide identity for the complete L segments, 99.94% to 99.97% nucleotide identity for the complete M segments, and 99.94% to 100.00% nucleotide identity for the complete S segments. The five samples isolated SFTSV strains were tested by the real-time PCR and the PCR products were sequenced by using plasmid cloning technique. The results show that 4 PCR products were sequenced successfully. The nucleotide sequences of PCR products were 100% similar to SFTSV isolated strains in this study (Table 2).

**Fig 1:**
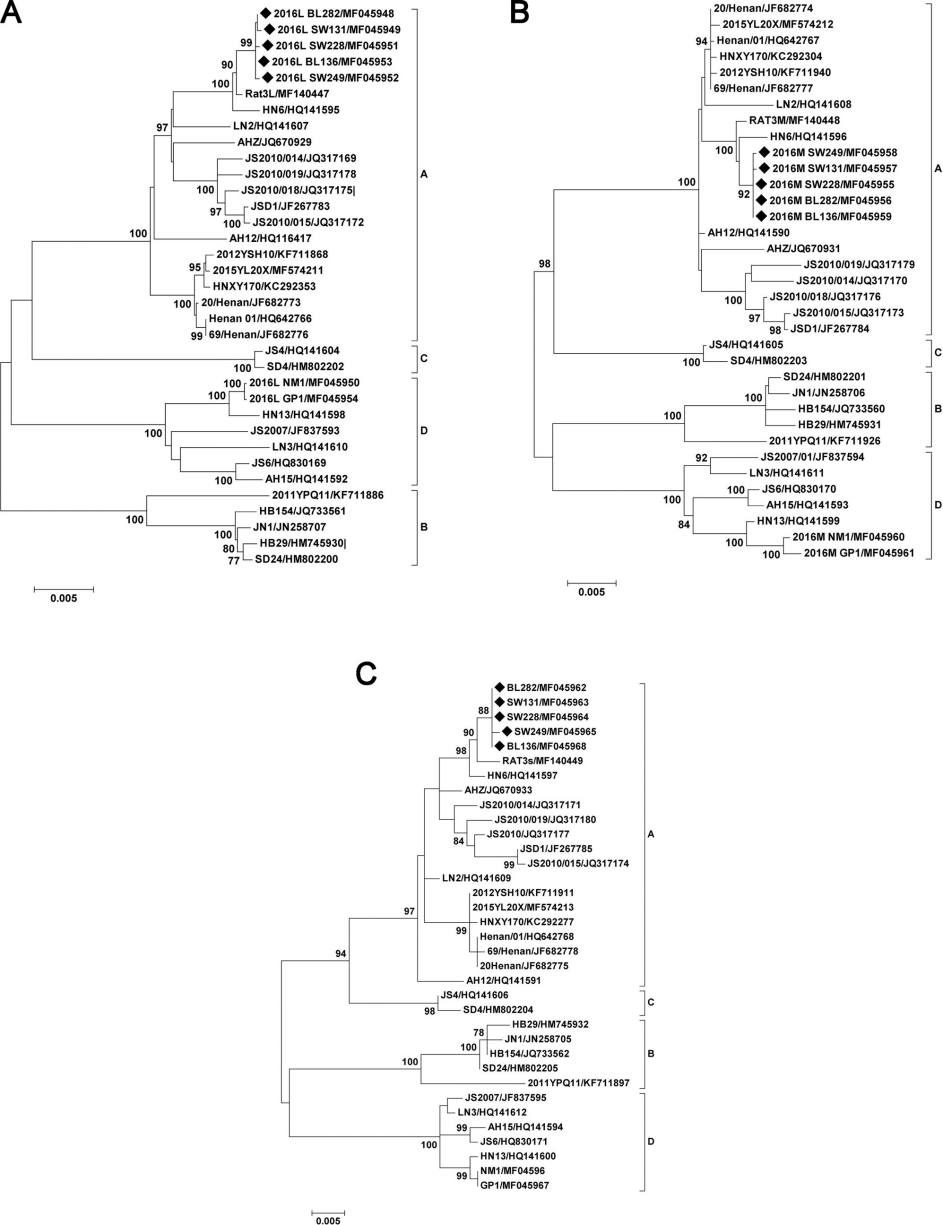
Phylogenetic analysis of SFTSV strains isolated from healthy people from Xinyang, Henan compared with other SFTSV. The phylogenetic tree was constructed by the maximum likelihood method with the MEGA5 software. The reliability values indicated at the branch nodes were determined using 1,000 bootstrap replications. Isolated SFTSV strains in this study were labeled by black solid diamonds. Phylogenetic relationship of SFTSV with other bunyaviruses, based on the complete L, M, S segment sequences, are shown in panel (A), (B), (C), respectively.

Phylogenetic analyses were performed using these five sequences as well as 30 additional full-length SFTSV sequences downloaded from GenBank and representing virus from different regions and times. The analysis revealed four major sub-lineages, referred to as genotypes A, B, C, and D [12], and showed that the L, M, and S sequences from the five isolates obtained in this study belonged to SFTSV genotype A (Fig 1).

## Discussion

SFTSV is an expanding global public health threat due to its high mortality and its increased incidence in several countries around the world [21,22]. A recent report showed that an estimated 8.3% of SFTS cases went undiagnosed in high endemic areas [23], possibly because the cases lacked typical clinical features of disease, such as fever or thrombocytopenia. If true, these data suggest that the actual incidence of SFTS may be much higher than currently reported. Therefore, in an effort to better understand the dynamics of STFTS, we performed a cross-sectional study to evaluate the seroprevalence of SFTSV-specific antibodies in healthy individuals in Xinyang rural region.

In this study, we investigated 1463 healthy persons in 14 natural villages of Xinyang city from April to May 2016. The average seropositive rate of SFTSV-specific IgG was 10.46% (153/1463). To our surprise, 12 individuals were positive for IgM, and six of these were also positive for SFTSV RNA, suggesting that these people were experiencing an active SFTSV infection. Indeed, we isolated SFTSV from five of the six people. Interestingly, upon a one-month follow-up visit, none of the 12 individuals reported having experienced any symptoms consistent with SFTS (although we cannot rule out the confounding role that recall bias may have played). To our knowledge, this is the first report of the isolation of SFTSV from otherwise healthy individuals, and it suggests the existence of asymptomatic SFTSV infections, which may have significant effects on the dynamics of SFTS outbreaks. Future work should focus on the underlying factors that contribute to the development of asymptomatic SFTSV infections, such as host genetic background [24], as well as the degree to which asymptomatic cases may infect other individuals. Indeed, additional control measures may be necessary in high endemic regions to prevent asymptomatic individuals from propagating SFTS outbreaks.

The predominant cause of the epidemic in this area. A previous Bayesian evolutionary analysis suggested that SFTSV likely originated 50–225 years ago in areas around Dabie mountain, which borders Henan, Anhui and Hubei provinces [25]. The majority of the SFTSV strains from mainland China belong to genotypes A, B and D, while genotype C is less frequent and mainly found in Jiangsu and Shandong provinces [26]. Although genotypes A, B, and D circulate in Henan province, genotype A has always been predominant [12]. Considering the predominance of this genotype in Henan province, as well as the fact that all participants from whom SFTSV was isolated lived in the same town (Balifan), it is not surprising that the nucleotide sequences of SFTSV isolates were highly homologous, and belonged to genotype A.

Previous epidemiologic studies have examined the seroprevalence of SFTSV-specific antibodies among healthy individuals in China and have reported seropositive rates ranging from 0.23% to 9.17%, depending on the region surveyed [27]. Indeed, a previous study in Xinyang city—the same geographical regions surveyed in the present study—determined a seropositive rate of 6.59% from 2011 to 2013 [28]. This value is slightly less than ours and may suggest that the incidence of SFTS is increasing in the area, perhaps due to the increased distribution of SFTSV or increased exposure of people to the virus [29]. Notably, however, past seroprevalence investigations have been limited by small sample sizes or survey locations in urban (as opposed to rural) areas, and sampling methods may differ between studies, thus limiting comparisons.Further research is therefore required to continue to improve our understanding of SFTS in China and elsewhere.

## Supporting information

S1 Checklist STROBE statement—Checklist of items that should be included in reports of observational studies. (DOC)
